# Systemic interleukin-6 inhibition ameliorates acute neuropsychiatric phenotypes in a murine model of acute lung injury

**DOI:** 10.1186/s13054-022-04159-x

**Published:** 2022-09-13

**Authors:** Faizan Anwar, Nicklaus A. Sparrow, Mohammad Harun Rashid, Gena Guidry, Michael M. Gezalian, Eric J. Ley, Maya Koronyo-Hamaoui, Itai Danovitch, E. Wesley Ely, S. Ananth Karumanchi, Shouri Lahiri

**Affiliations:** 1 Department of Neurology, Cedars-Sinai Medical Center, Los Angeles, CA USA; 2 Department of Neurosurgery, Cedars-Sinai Medical Center, Los Angeles, CA USA; 3 Division of Trauma and Critical Care, Department of Surgery, Cedars-Sinai Medical Center, Los Angeles, CA USA; 4 Division of Applied Cell Biology and Physiology, Department of Biomedical Sciences, Cedars-Sinai Medical Center, Los Angeles, CA USA; 5 Department of Psychiatry and Behavioral Neurosciences, Cedars-Sinai Medical Center, Los Angeles, CA USA; 6 Critical Illness, Brain Dysfunction, Department of Pulmonary and Critical Care Medicine, Survivorship (CIBS) Center, Veteran’s Affairs Tennessee Valley Geriatric Research Education and Clinical Center (GRECC), Vanderbilt University School of Medicine, Nashville, TN USA; 7 Department of Medicine, Cedars-Sinai Medical Center, Los Angeles, CA USA; 8 Departments of Neurology, Neurosurgery and Biomedical Sciences, Cedars-Sinai Medical Center, 8700 Beverly Blvd., Los Angeles, CA 90048 USA

**Keywords:** VILI, Delirium, Anxiety, IL-6, Neural injury, Neuroinflammation, Cleaved caspase-3

## Abstract

Acute neuropsychiatric impairments occur in over 70% of patients with acute lung injury. Mechanical ventilation is a well-known precipitant of acute lung injury and is strongly associated with the development of acute delirium and anxiety phenotypes. In prior studies, we demonstrated that IL-6 mediates neuropathological changes in the frontal cortex and hippocampus of animals with mechanical ventilation-induced brain injury; however, the effect of systemic IL-6 inhibition on structural and functional acute neuropsychiatric phenotypes is not known. We hypothesized that a murine model of mechanical ventilation-induced acute lung injury (VILI) would induce neural injury to the amygdala and hippocampus, brain regions that are implicated in diverse neuropsychiatric conditions, and corresponding delirium- and anxiety-like functional impairments. Furthermore, we hypothesized that these structural and functional changes would reverse with systemic IL-6 inhibition. VILI was induced using high tidal volume (35 cc/kg) mechanical ventilation. Cleaved caspase-3 (CC3) expression was quantified as a neural injury marker and found to be significantly increased in the VILI group compared to spontaneously breathing or anesthetized and mechanically ventilated mice with 10 cc/kg tidal volume. VILI mice treated with systemic IL-6 inhibition had significantly reduced amygdalar and hippocampal CC3 expression compared to saline-treated animals and demonstrated amelioration in acute neuropsychiatric behaviors in open field, elevated plus maze, and Y-maze tests. Overall, these data provide evidence of a pathogenic role of systemic IL-6 in mediating structural and functional acute neuropsychiatric symptoms in VILI and provide preclinical justification to assess IL-6 inhibition as a potential intervention to ameliorate acute neuropsychiatric phenotypes following VILI.

**Supplementary Information:**

The online version contains supplementary material available at 10.1186/s13054-022-04159-x.

## Introduction

Acute neuropsychiatric impairments, such as delirium and anxiety, occur in over 70% of patients with acute lung injury [1–3]. Acute lung injury is a multi-etiological condition that has recently garnered notoriety as a common complication of severe acute respiratory syndrome coronavirus-2 (SARS-CoV-2), occurring in over one-third of patients who contract the virus [4]. While mechanical ventilation is frequently used to treat patients with acute lung injury, it is also a well-known precipitant of acute lung injury, i.e., ventilator-induced lung injury (VILI), and an established risk factor for acute neuropsychiatric phenotypes [5, 6].

In prior studies, we demonstrated a key pathological role for systemic interleukin-6 (IL-6) in mediating frontal and hippocampal structural neuronal injury in a murine model of VILI [7]; however, what role this cytokine plays in mediating acute neuropsychiatric phenotypes remain unknown. Although various VILI-associated clinical risk factors, such as duration of mechanical ventilation and use of sedatives, have been associated with acute neuropsychiatric symptoms [3, 8], the specific underlying biological mechanisms are not known. It has been hypothesized that, in the general population, immune-mediated injury to the amygdala and hippocampus plays a central role in the pathogenesis of acute neuropsychiatric states, specifically by the cytokines IL-6, IL-1β, and TNF-α [9–16]. Increased IL-6 signaling has been associated with a diversity of neuropsychiatric impairments including delirium [17–19] and is frequently elevated in individuals with acute encephalopathy and VILI [20, 21]. We previously demonstrated elevated cortical IL-6 in response to VILI [7]; however, it is not known whether the acute inflammatory response of VILI contributes to acute neuropsychiatric impairments, such as delirium- and anxiety-like behaviors.

In this study, we hypothesized that a murine model of VILI [22] would demonstrate evidence of immune-mediated structural neural injury as well as functional impairments of the amygdala and hippocampus. Furthermore, as systemic IL-6 inhibition has been demonstrated to ameliorate VILI-induced neural injury [23–26], we hypothesized that systemic IL-6 inhibition would ameliorate both the neural injury and functional impairments of the amygdala and hippocampus, brain regions that are implicated in the pathogenesis of acute neuropsychiatric impairments.

## Material and methods

### Mice

A total of fifty-four male or female C57BL/6 mice aged 6–7 months old (Jackson Laboratory) were used in this study. Animals were housed in Cedars-Sinai’s AAALAC accredited animal facility under standard conditions (kept in ventilated cages at approximately 21 °C, 40–70% humidity, a 12-h light/dark cycle, with food and water available to the animals ad libitum). All procedures and behavioral testing were approved by Cedars-Sinai’s IACUC (protocol # IACUC007914) prior to any work commencing and entirely new cohorts of animals were used for this study. Based on preliminary immunohistochemical analysis using CC3 (cleaved caspase-3), the greatest group SD and mean difference were 0.12 and 0.3, respectively. Using these values, a power analysis with one-way ANOVA and Tukey’s post hoc test yielded 0.90% power at the 0.05 significance level with *n* = 5/group. The average attrition rate across experiments was 5.8%. Attrition was exclusively due to associated complications after extubation, and each instance of mortality, the mouse was replaced in a subsequent experiment.

#### Model establishment/hypothesis generation

We randomized mice to one of the following groups: mice anesthetized and mechanically ventilated with 35 cc/kg tidal volume (VILI) (*n* = 6), mice anesthetized and mechanically ventilated with 10 cc/kg tidal volume (*n* = 6), or spontaneously breathing (SB) mice (*n* = 6).

#### Hypothesis validation

To examine the effects of IL-6 inhibition on the neuropathology of brain regions implicated in acute neuropsychiatric impairments, mice were randomized to one of the following groups: VILI + Saline (*n* = 5), VILI + anti (α)-IL-6 antibody (*n* = 6), VILI + α-IL-6-receptor (*n* = 6), or SB controls (*n* = 5).

### VILI model

To induce VILI in mice, we utilized a previously validated model of VILI where we subjected fifty-four mice to high tidal volume (35 cc/kg) mechanical ventilation for two hours [22, 27–29]. In the mechanically ventilated mice, severe lung injury is induced by mechanically stretching the alveolar walls. The stretching of the alveolar wall causes cell deformation, endothelial and epithelial layer breakdown, interstitial edema, and produces inflammatory infiltrates that can be measured in the bronchoalveolar lavage fluid (BALF) [28]. Histological evidence of acute lung injury using this model has been demonstrated in our prior publication [7].

To rule out possible effects of anesthesia and mechanical ventilation on neural injury and inflammation, we provided the same anesthesia regimen and mechanically ventilated mice with 10 cc/kg [30–32]. Mice subjected to VILI or those mechanically ventilated with 10 cc/kg tidal volume were first anesthetized with intraperitoneal injection of a mix of ketamine (Vedco Inc.) and dexmedetomidine (Pfizer) (75 mg/kg and 0.5 mg/kg, respectively), then orotracheally intubated, and lastly mechanically ventilated using an Inspira volume-controlled small animal ventilator (Harvard Apparatus) in ambient, room air. To induce VILI, mice were subjected to the following mechanical ventilation parameters: a tidal volume of 35 cc/kg at a respiratory rate of 70 breaths per minute with zero positive end-expiratory pressure for a duration of 2 h. The mechanical ventilation parameters for the control 10 cc/kg group were as follows: a tidal volume of 10 cc/kg at a respiratory rate of 105 breaths per minute with zero positive end-expiratory pressure for a duration of 2 h. Subcutaneous saline (0.5 mL) was administered to maintain hydration, and the eyes of mice were protected with a thin coat of Paralube (Dechra) immediately before intubation. During mechanical ventilation, the body temperature of mice was maintained using a 38 °C heating pad (Hallowell EMC). Anesthesia was reversed with atipamezole (1 mg/kg in 100 μL of sterile water), and the mice were allowed to recover in their cages on a heating pad for 4 h before euthanasia followed by tissue collection. Control mice were euthanized together with the VILI mice.

### IL-6 inhibition

IL-6 signaling was inhibited by systemic administration of two monoclonal antibodies: one which binds IL-6 peptide (α-IL-6) and another that binds the IL-6 receptor (α-IL-6R) (Bio *X* Cell α-IL-6, clone MP5-20F3; α-IL-6R, clone 15A7). Each antibody-treated mouse received 200 µg of either antibody as a 500µL intraperitoneal injection of a 0.4 ug/µL solution in saline. The dosing ranged from 7.4–9.4 mg/kg, and the average dose was 8.4 mg/kg. Control mice (VILI + Saline) received 500 µL of saline only. After intraperitoneal administration of the IL-6 inhibitors, mice were then placed back into cages for 2 h to allow maximum serum concentrations to be reached [33] after which VILI was induced as outlined in the *VILI Model* section above. During ventilation, oxygen saturation was recorded using the MouseOx^®^ Plus (v. 1.6; Starr Life Sciences Corp.) system with the thigh sensor.

### Brain isolation and treatment

Mice were deeply anesthetized and perfused with room temperature PBS with 0.5 mM ethylenediaminetetraacetic acid (10 mL). Right hemispheres were collected and fixed by submerging in ice-cold PBS buffered 4% paraformaldehyde (Electron Microscopy Sciences) for 30 min, and then cryo-protected in 4% paraformaldehyde + 30% sucrose at 4 °C for 24 h. Free-floating, 30-μm-thick coronal brain cryosections were prepared and stored at 4 °C in PBS + 0.02% sodium azide until staining. Mouse tissue obtained from an acute ischemic stroke mouse model was used as the positive control for hypoxic neural death [34].

### Immunohistochemistry (IHC) and microscopy

Sections were affixed to slides by air drying and subjected to heat-induced epitope retrieval for 10 min in antigen-retrieval solution (pH 6.0; Invitrogen) prior to permeabilization/blocking in 5% BSA + 0.25% Triton X-100 in PBS for 1 h at room temperature. Sections were then incubated at 4 °C overnight with primary antibodies diluted in 1% BSA + 0.01% Triton X-100 in PBS (Ab Diluent) (See Additional file 1: Table S1 for antibody information). After washing, sections were incubated with a combination of the appropriate secondary antibody (Alexa Fluor Plus conjugated; Invitrogen) diluted to 4 µg/mL in Ab Diluent for 1 h at room temperature. After washing, sections were incubated in 0.05% Sudan black B in 70% ethanol for 10 min to reduce tissue autofluorescence. Sections were mounted using ProLong Glass with DAPI (Invitrogen). Negative controls were processed using the same protocol with the omission of the primary antibody to assess non-specific labeling. A Carl Zeiss AxioImager Z.2 epi-fluorescence microscope—equipped with standard filter sets/mercury arch lamp, an Apotome 2.0, and an Axiocam HRm camera—controlled by Zen Blue Pro (version 2.3) software was used to acquire and process images. Images of damage marker (e.g., cleaved caspse-3 (CC3)) staining were acquired with a 10 × objective (NA 0.3, Zeiss) as a 3 × 3 tiled image that encompassed the amygdala and a 5 + 5 tile image that encompassed the hippocampus of each section. Images of cytokines (IL-6, IL-1β, and TNF-α) staining were acquired with the Apotome 2.0 and a 20 × objective (NA 0.8, Zeiss) as a single field, 8 µm z-stacks (1 µm interval), and were analyzed and displayed as maximum intensity projections. All acquisition and display settings are the same between groups, and settings were optimized using the VILI group. All images within a figure are set to the same scale.

CC3 was quantified as a potentially reversible neural injury marker that correlates with functional impairments induced by acute systemic disease processes [16, 22].

### Image and statistical analysis

Fiji (ImageJ v. 1.53c) software was used for image analysis and quantitation. Prism 9.0.1 (GraphPad) was used for statistical analysis. Three coronal sections (slices) containing the hippocampus and amygdala were analyzed (one ventral, one mid, and one dorsal) for each animal. For damage marker analysis, two different regions of interest (ROIs) were drawn on tiled images of each section: a ROI around the amygdala, or a ROI encompassing the entire hippocampus (both with an average area of 250 µm^2^). A threshold was set to exclude background pixels per ROI based on the pixel intensity histogram and the number of positive pixels was measured and then expressed as percent area of the ROI. For cytokine analysis, a single-field z-stack projection from the amygdala and hippocampus was analyzed per section. Background pixels were excluded per field based on the pixel intensity histogram and the intensity of the remaining pixels was used to calculate percent area. Values for each protein from the triplicate sections were averaged to yield one number per animal. Analysis was performed by assessors blinded to group allocation. Statistically significant outliers were determined (ROUT method with a *Q* = 10%) and excluded, and either one-way analysis of variance (ANOVA) with multiple comparisons (Tukey’s) or unpaired Welch’s t test was used to determine statistical significance between groups. Simple linear regression analyses were utilized to examine relationships between variables. For all figures, quantitative data were expressed as mean ± standard deviation (SD) unless otherwise stated. Differences with *p* ≤ 0.05 were considered significant (* = *p* ≤ 0.05, ** = *p* ≤ 0.01, *** = *p* ≤ 0.001, **** = *p* ≤ 0.0001).

### Behavioral testing

A cohort of fourteen 6–7-month-old mice were used for behavioral testing. VILI was induced in all animals—7 were treated with α-IL-6 antibody and 7 were treated with saline as described above in *VILI model* and *IL-6 inhibition*. Behavioral testing was limited to VILI animals to account for differences in locomotor activity induced by acute lung injury and performed 8 h after inducing VILI to allow for sufficient recovery from anesthetic and acute lung injury effects. Further, behavioral testing was limited to female mice only, informed by our experience that male mice with VILI do not participate with the behavioral testing assays.

### Open field

Locomotor activity, delirium-, and anxiety-related emotional behaviors were evaluated by placing mice individually into the center of a clear Plexiglas (40 × 40x40 cm) open-field arena and allowing the animals to explore for 45 min [35–37]. Activity in the open field was recorded by a computer-operated camera system. Center distance (the distance traveled in the center of the arena) and center time (the time spent in the center of arena) were analyzed by ANY-maze video tracking software version 7.00 (Stoelting Co., Wood Dale, IL, USA). The open field was divided into a 20 × 20 cm central zone and a surrounding border zone during the analysis. Data were collected over the 45-min test session.

### Elevated plus maze

Delirium- and anxiety-like behavior was assessed using elevated plus maze [36, 38, 39]. The elevated plus maze crossed arms were approximately 30 cm above the floor during testing, with open and closed arms crossed perpendicularly to each other. A video camera was placed above the apparatus and ANY-maze video tracking software 7.00 (Stoelting Co., Wood Dale, IL, USA) was used to analyze the behavior. At the start of the behavior, the mouse was placed in the center of the crossed arms facing an open arm and allowed to freely explore the entire maze for 5 min. Percent entries in open arms and percent time spent in the open arms were recorded. The percent entries or time spent in open arm was calculated by dividing total entries or time spent in open arm by sum of entries or time spent in open arms and closed arms.

### Y-maze

The Y-maze test was used to assess attentional, cognitive, and short-term memory deficits in VILI mice [36, 40]. Each arm of the maze was labeled as either A, B, or C. In each session, the animal is placed in arm A and allowed to explore the three arms for 5 min. Activity in the Y-maze was recorded by a computer-operated video recording system. Number of arm entries are scored from the recorded video file to calculate the percent alternation. The alternation percentage is calculated by dividing the number of alternations by number of possible triads × 100. The elevated plus maze and Y-maze were cleaned with ethanol solution between animals to eliminate odor traces.

## Results

**Fig. 1:**
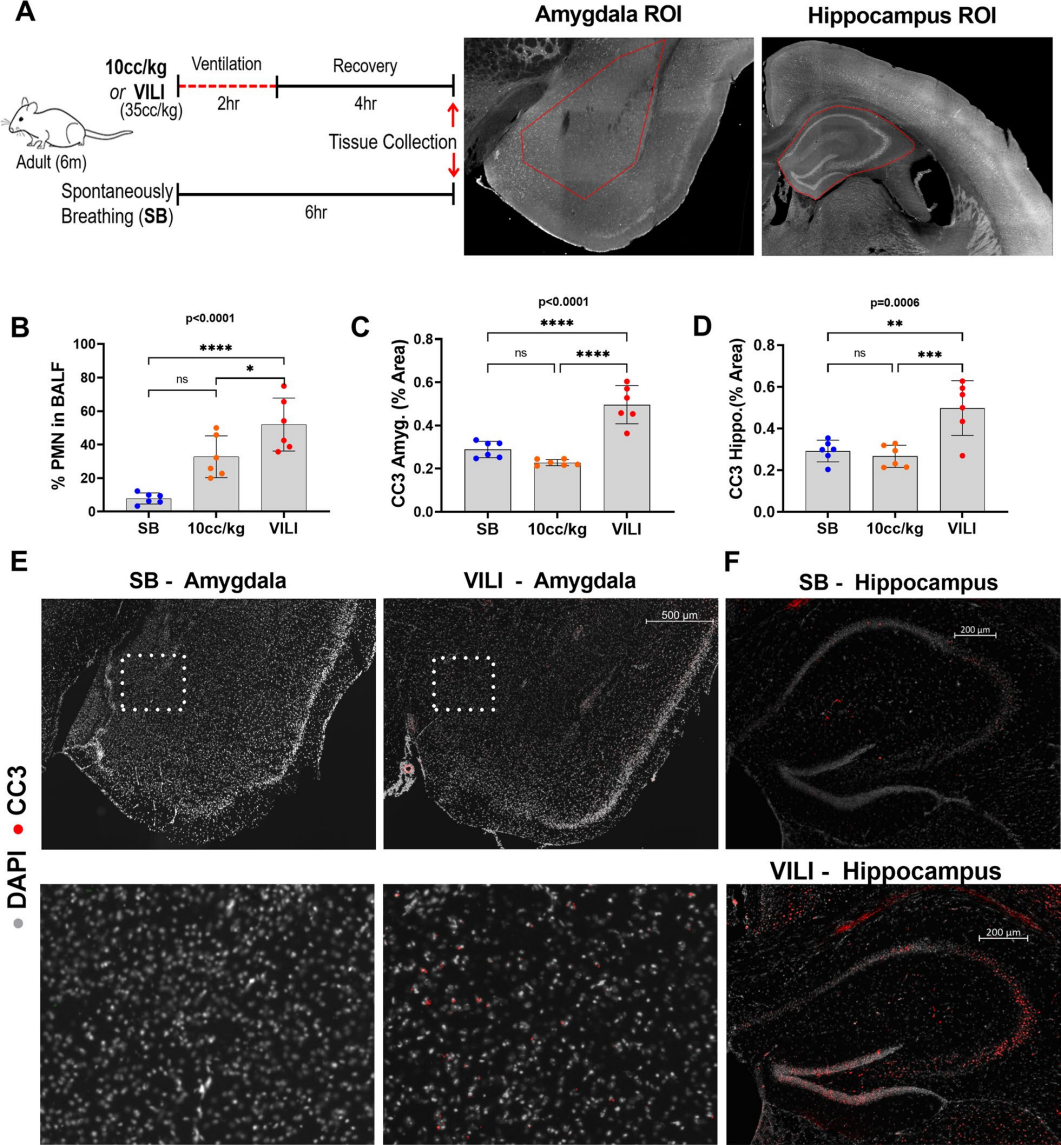
Cleaved caspase-3 is significantly elevated in the amygdala and hippocampus of VILI mice. **A** Schematic of experimental design and representative regions of interest (ROIs) of measured brain regions. **B** Quantification of lung inflammation (%PMN in BALF). **C–D** Quantification of cleaved caspase-3 (CC3) percent area in the amygdala and the hippocampus where each dot represents one animal. **E** Representative amygdalar sections stained for CC3 (positive signal displayed in red) overlaid on DAPI nuclear stain (gray). Magnified regions of the indicated area of amygdala from the micrograph directly above. **F** Representative hippocampal sections stained for CC3 (positive signal displayed in red) overlaid on DAPI nuclear stain (gray). Quantitative data are expressed in mean ± SD. *p ≤ 0.05, **p ≤ 0.01, ***p ≤ 0.001, ****p ≤ 0.0001

The timeline of the experimental design and representative amygdalar and hippocampal brain regions used for analysis are shown in Fig. 1A. There were no significant differences between sex, age, and weight of the mice between the groups.

## Hypothesis generation

### VILI induces neural injury and neuroinflammation

Figure 1B shows an increase in lung inflammation, measured by the percent of polymorphonuclear (PMN) cells in BALF, in the VILI group compared to controls who were either SB or anesthetized and mechanically ventilated with 10 cc/kg (overall ANOVA *p* ≤ 0.001). Induction of apoptosis triggers multiple pathways that results in the cleavage of caspase-3 to CC3, which further activates downstream apoptotic pathways, ultimately culminating in fragmentation of the genome and cell death [41]. CC3 expression was significantly elevated in the amygdala and hippocampal brain regions of the VILI group compared to SB or 10 cc/kg control groups (overall ANOVA *p* ≤ 0.0001 and *p* = 0.0006 respectively; Fig. 1C–D), but there was no evidence of cell death indicated by the absence of terminal deoxynucleotidyl transferase DUTP nick end labeling (TUNEL)-positive staining in the VILI mice (Additional file 1: Fig. S1). Furthermore, to assess any effects of VILI-induced cerebral hypoxic injury, we analyzed the expression of hypoxia-inducible factor-1α (HIF-1α) protein, a highly sensitive transcription factor that is up-regulated in response to hypoxia. There was no evidence of increased expression of HIF-1α in the amygdala of mice with VILI (Additional file 1: Fig. S1). Representative micrographic images of CC3 staining in the amygdala and hippocampus are shown in Fig. 1E–F.

**Fig. 2:**
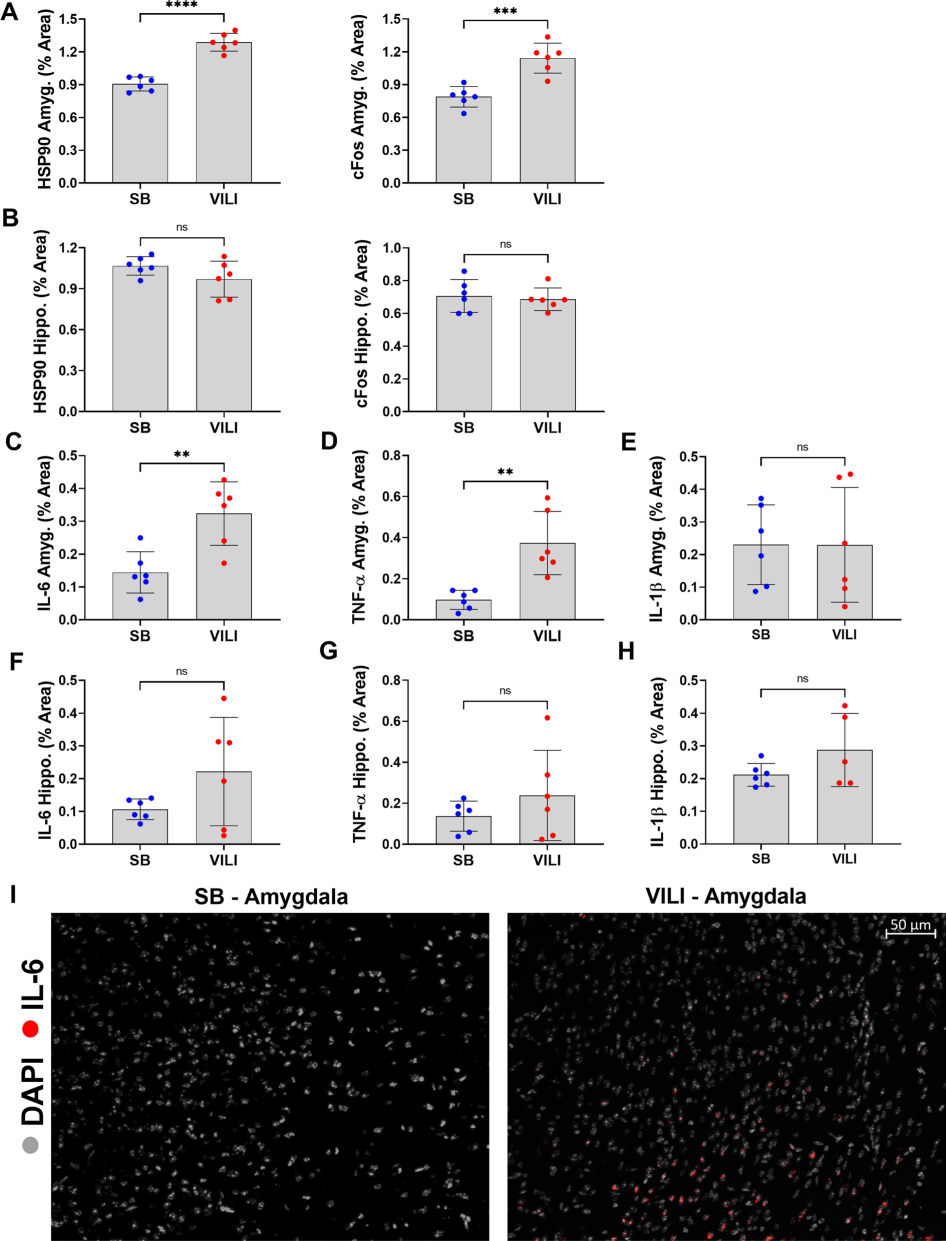
VILI increases amygdalar neuronal activity, neuronal stress response, and both amygdalar and hippocampal IL-6 and TNF-α. **A–B** Quantified levels of cellular stress response (HSP90) and of neuronal activity (*c-fos*) within the amygdala and hippocampus. **C–H** IL-6 and TNF-α are significantly increased in VILI brains compared to SB controls in amygdala but not in the hippocampus, while there is no significant change in IL-1β in both areas**. I** Representative micrographs stained for IL-6 (positive signal displayed in red); cell nuclei are revealed by DAPI staining (gray). Quantitative data are expressed in mean ± SD. *p ≤ 0.05, **p ≤ 0.01, ***p ≤ 0.001, ****p ≤ 0.0001

To assess neural activity and stress, we quantified the expression of *c-fos* and the chaperone protein heat-shock protein-90 (HSP90), respectively. There was an approximately 1.4-fold increase in both *c-fos* and HSP90 in the amygdala of VILI mice compared to SB (*p* = 0.0006 and *p* ≤ 0.0001, respectively; Fig. 2A); however, there were no differences in expression of these proteins within the hippocampus after induction of VILI (Fig. 2B).

To identify the potential roles of cytokine-mediated changes on VILI-induced brain injury, we measured amygdalar and hippocampal expressions of inflammatory cytokines, IL-6, IL-1β, and TNF-α, which are implicated in the pathogenesis of acute neuropsychiatric impairments [16–18, 42–49]. Compared to SB, there was a 2.2-fold increase in IL-6 expression in the amygdala and an approximately 3.8-fold increase in amygdalar TNF-α levels (*p* ≤ 0.01 and *p* ≤ 0.01, respectively; Fig. 2C–D). There were no significant differences with IL-1β (Fig. 2E). There was an approximately twofold increase in IL-6 expression in the hippocampus (Fig. 2F); however, this observed increase in IL-6 approached but did not reach significance. Additionally, there were no significant changes in TNF-α or IL-1β in the hippocampus (Fig. 2G–H).

**Fig. 3:**
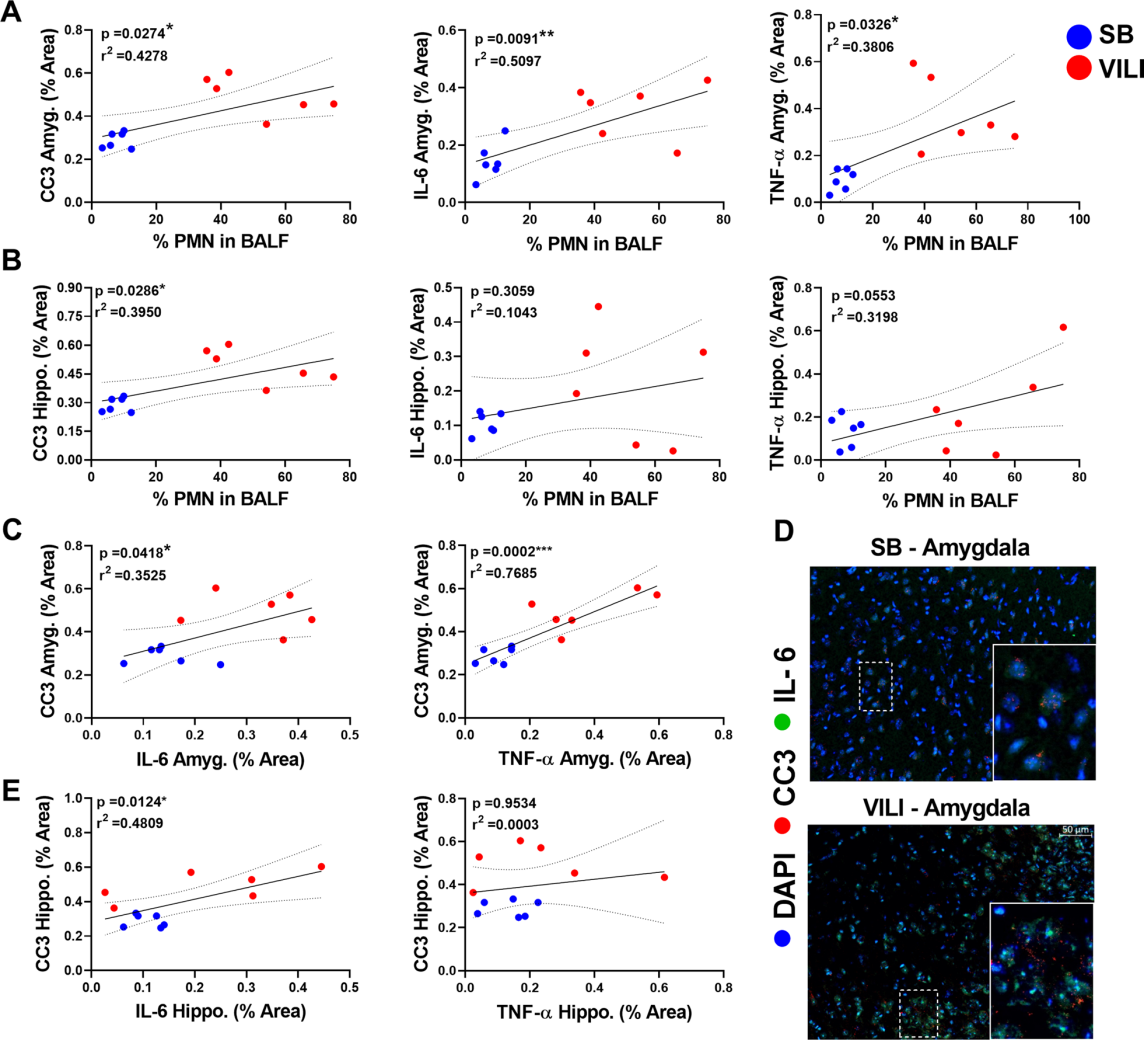
Lung inflammation, cerebral IL-6, and cerebral TNF-α positively and significantly correlate with both amygdalar and hippocampal CC3. **A–B** Regression analysis between the percent of PMNs in bronchoalveolar lavage fluid (BALF) with amygdalar and hippocampal CC3, IL-6, or TNF-α. SB group is indicated in blue and VILI in red. Significance (*p*) and fitness (*r*^2^) values are indicated. **C** Regression analysis demonstrating significant positive relationships between amygdalar CC3 with IL-6 and TNF-α. **D** Micrographs of amygdalar cortical neurons stained for IL-6 (displayed in green) and CC3 (displayed in red). Magnified areas of individual neurons and glial cells are inset. Note that CC3-positive neurons also co-stain for IL-6 in VILI amygdala. **E** Regression analysis demonstrates a significant positive relationship between hippocampal CC3 with IL-6 but not with TNF-α

PMNs in the BALF were positively and significantly correlated with CC3, IL-6, and TNF-α expression in the amygdala (*r*^2^ = 0.4278/*p* = 0.0274, *r*^2^ = 0.5097/*p* = 0.0091, & *r*^2^ = 0.3806/*p* = 0.0326, respectively; Fig. 3A), while there was no significant relationship with IL-1β (Additional file 1: Fig. S2A). There were positive and significant associations between percent PMNs in the BALF with CC3 (*r*^2^ = 0.3950/*p* = 0.0286; Fig. 3A), but no significant correlations with IL-6 and TNF-α (Fig. 3B, C) or IL-1β (Additional file 1: Fig. S2B) in the hippocampus.

Notable associations were found between brain inflammatory cytokines and CC3 in the amygdala: amygdalar IL-6 or TNF-α were significantly and positively correlated with CC3 (*r*^2^ = 0.3525/*p* = 0.0418 & *r*^2^ = 0.7685/*p* = 0.0002, respectively; Fig. 3C); however, there was no significant correlation between IL-1β and amygdalar CC3 (Additional file 1: Fig. S2A). Furthermore, representative micrographs demonstrated an apparent colocalization between IL-6 and CC3-positive neurons in amygdala (Fig. 3D). A significant relationship between hippocampal CC3 expression and IL-6 was identified (*r*^2^ = 0.4809/*p* = 0.0124; Fig. 3E) but not with TNF-α (*r*^2^ = 0.0003/*p* = 0.9534; Fig. 3E). Additionally, a significant association between CC3 and IL-1β (*r*^2^ = 0.5267/*p* = 0.0115; Additional file 1: Fig. S2B) was found in the hippocampus.

### Systemic IL-6 inhibition ameliorates amygdalar and hippocampal CC3

**Fig. 4:**
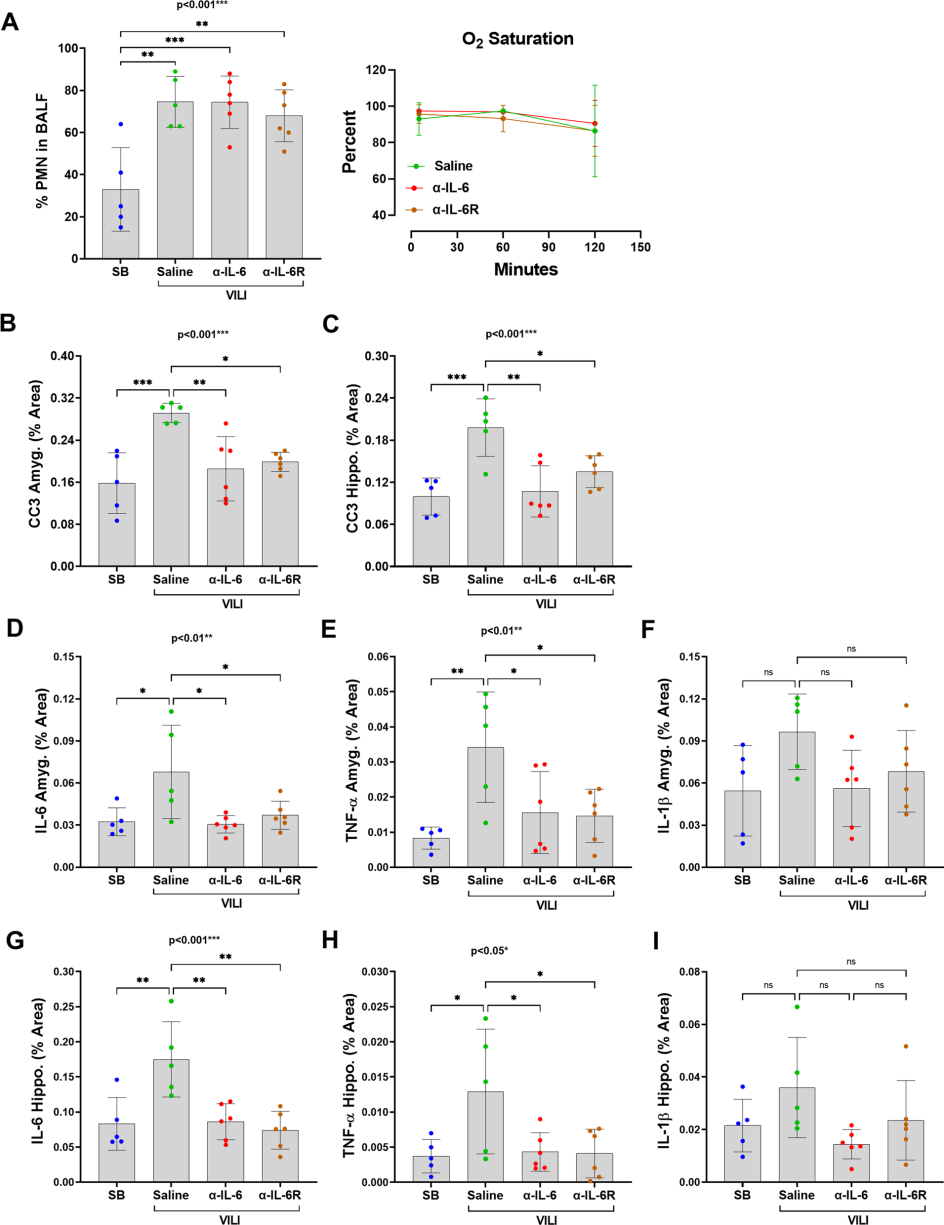
IL-6 inhibition significantly reduces amygdalar or hippocampal CC3 expression. **A** PMNs in BALF after VILI were significantly different from SB regardless of the three intervention groups (ANOVA, *F* = 9.861, dF = 21, *p* = 0.0005). There are no significant differences in oxygen saturations between the three VILI intervention groups; VILI + Saline, VILI + α-IL-6, and VILI + α-IL-6-receptor antibody. **B–C** Amygdalar (ANOVA, *F* = 8.738, dF = 21, *p* = 0.0009) and hippocampal (ANOVA, *F* = 9.806, dF = 21, *p* = 0.0005) CC3 expressions are significantly increased in the VILI + Saline group compared to SB control, but significantly reduced in both IL-6 inhibited groups. **D–E** IL-6 (ANOVA, *F* = 5.160, dF = 21, *p* = 0.0095) and TNF-α (ANOVA, *F* = 5.655, dF = 21, *p* = 0.0066) are significantly increased in the VILI + Saline group compared to SB control, but significantly reduced in both VILI + IL-6 inhibited groups. **F** There is no significant difference in percent area of amygdalar IL-1β between all groups. **G–I** Similar to amygdalar cytokines, IL-6 (ANOVA, F = 8.658, dF = 21, *p* = 0.0009) and TNF-α (ANOVA, *F* = 4.210, dF = 21, *p* = 0.0202) are significantly increased in the VILI + Saline group compared to SB control and significantly reduced in both VILI + IL-6 inhibiter groups; however, there is no difference in the percent area of IL-1β between all groups

To determine possible pathophysiological implications of IL-6 signaling inhibition on structural phenotypes of acute neuropsychiatric impairment, we examined the effects of inhibition of either IL-6 or IL-6-receptor (IL-6R) in mice subjected to VILI [50–52]. Percent of PMNs was significantly increased in all three VILI compared to SB; however, there were no significant differences in percent PMNs or oxygen saturation between the VILI groups (Fig. 4A). Amygdalar and hippocampal CC3 expression in α-IL-6- or α-IL-6R-treated VILI groups were significantly reduced compared to saline-treated VILI mice (Fig. 4B–C). There were corresponding significant changes in amygdalar and hippocampal IL-6 and TNF-α, and a nonsignificant trend toward decreased IL-1β expression in the IL-6 inhibited compared to the saline-treated groups in both brain regions (Fig. 4D–I).

**Fig. 5:**
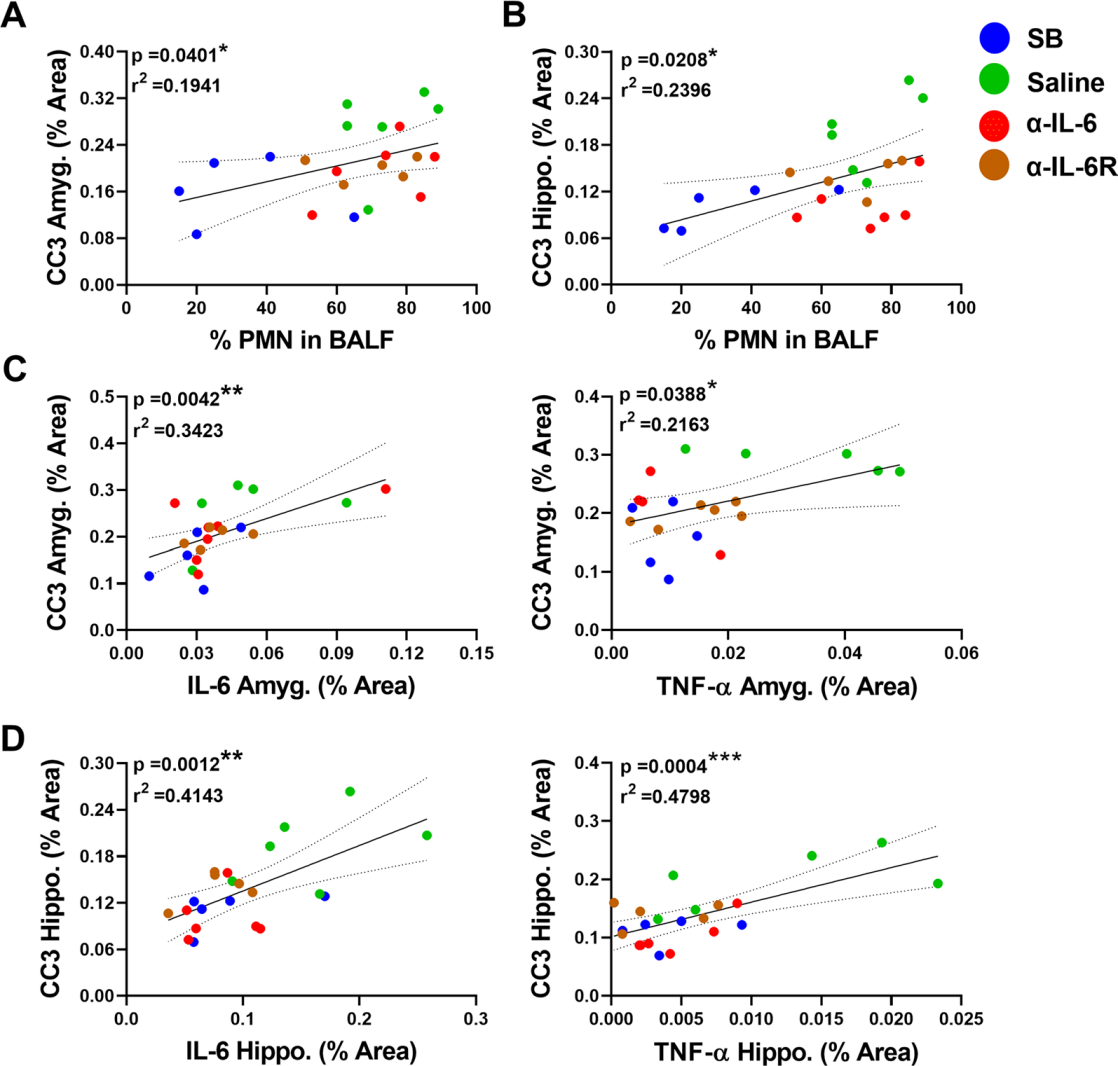
Lung inflammation, cerebral IL-6, and cerebral TNF-α positively and significantly correlate with amygdalar and hippocampal CC3 after IL-6 inhibition. **A** Regression analysis between the percent of PMNs in bronchoalveolar lavage fluid with amygdalar CC3. SB group is indicated in blue and VILI + Saline in green, VILI + α-IL-6 in red, and VILI + α-IL-6-receptor in brown. Significance (*p*) and fitness (*r*^2^) values are indicated. **B** Regression analysis between the percent of PMNs in bronchoalveolar lavage fluid with hippocampal CC3. **C–D** Regression analysis shows a direct and significant relationship between IL-6 and CC3, and TNF-α and CC3 in amygdala and hippocampus

Positive associations were found between percent PMNs in the BALF and CC3 expression in the brain in both the amygdalar and hippocampal regions (*r*^2^ = 0.1941/*p* = 0.0401 & *r*^2^ = 0.2396/*p* = 0.0208, respectively, Fig. 5A–B), while no significant correlations were found between inflammatory cytokines in the brain and percent PMNs in the BALF (Additional file 1: Fig. S2C). Additionally, there were significant relationships between amygdalar CC3 and IL-6 (*r*^2^ = 0.3423/*p* = 0.0042), amygdalar CC3 and TNF-α (*r*^2^ = 0.2163/*p* = 0.0388), hippocampal CC3 and IL-6 (*r*^2^ = 0.4143/*p* = 0.0012), and hippocampal CC3 and TNF-α (*r*^2^ = 0.4798/*p* = 0.0004; Fig. 5C–D); however, no significant associations were found between CC3 and IL-1β within the same regions (Additional file 1: Fig. S2D). Because IL-6 upregulates a complex signaling pathway, we also examined amygdalar and hippocampal CC3 expression in mice administered α-IL-6 immediately after the completion of ventilation and noted a partial response (Additional file 1: Fig. S3), indicating that early treatment is necessary for the observed effect.

### Systemic IL-6 inhibition ameliorates delirium- and anxiety-like behavioral phenotypes

**Fig. 6:**
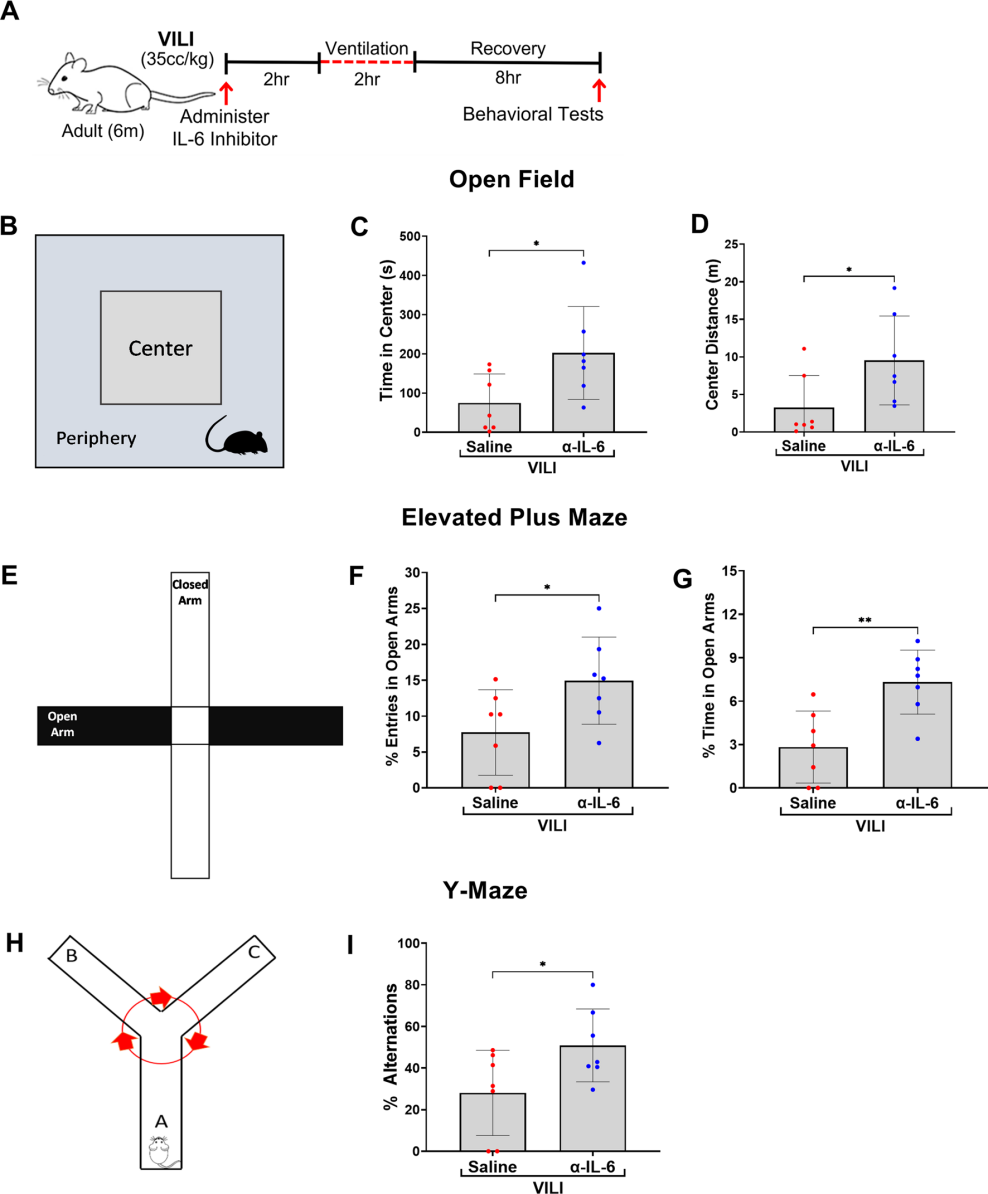
Systemic IL-6 inhibition significantly improves acute delirium- and anxiety-like neuropsychiatric functional impairments following VILI. **A** Schema of the apparatus and assessment of behavioral function in the center and peripheral zones of the Open Field. **B–C** In the open-field behavioral test, VILI + Saline mice spent significantly less time and traveled less distance in the center zone compared to the VILI + α-IL-6 group, indicating more anxiety behavior in the VILI + Saline group compared to the α-IL-6 treated group. **D** Schema of the elevated plus maze for behavioral assessment in open and closed arms. **E–F** In the elevated plus maze, the VILI + Saline mice entered and spent significantly less time in the open arms as compared to VILI + α-IL-6 mice, indicating increased anxiety and avoidance behaviors in the VILI + Saline group. **G** Schema of the Y-maze paradigm. **H–I** VILI + Saline mice had significantly less spontaneous alternations in the Y-maze compared to the α-IL-6 treated group, indicating impaired short-term memory. Quantitative data are expressed in mean ± SD. *p ≤ 0.05, **p ≤ 0.01, ***p ≤ 0.001, ****p ≤ 0.0001

A total of three behavioral tests were used to assess acute neuropsychiatric behaviors: open field, elevated plus maze, and Y-maze [35–38, 53]. A schematic illustration of the experimental timeline of the behavioral tests is shown in Fig. 6A. The Open-Field test (Fig. 6B) was used as an assessment of anxiety, avoidance behavior, and negative mood alteration. Decreased time spent and distance covered in the center of the open field can be attributed to cognitive deficits, avoidance of trauma related stimuli, and negative alterations in mood, and are markers of PSTD-like behavior. Compared to VILI mice treated with α-IL-6 antibody, the VILI mice treated with saline spent significantly less time in the center area, indicating increased anxiety, avoidance, and mood alterations (*p* = 0.032, Fig. 6C). There was also significantly less distance covered in the center by the VILI + Saline group, compared to VILI + α-IL-6 group (*p* = 0.042, Fig. 6D). These changes were not associated with any differences in locomotor activity as measured by total distance traveled or total immobile time (Additional file 1: Fig. S4A–B). Elevated plus maze (Fig. 6E) was used as an additional assay of anxiety-related behavior [39, 40, 54–56]. Compared to the VILI + Saline-treated mice, the VILI + α-IL-6 mice entered more and spent more time in open arms suggesting less anxiety and avoidance like behavior[36] (*p* = 0.044 and *p* = 0.003 respectively, Fig. 6F–G). Additionally, Y-maze spontaneous alternation (Fig. 6H) was used to assess change in cognition, specifically short-term memory [57, 58]. Compared to VILI + α-IL-6 mice, VILI + Saline mice exhibited significantly less spontaneous alternations in the first 10 alternations that persisted for the entire five-minute duration of the Y-maze test indicating cognitive and short-term memory deficits (*p* = 0.045, Fig. 6I).

## Discussion

In this study, we demonstrate that systemic IL-6 inhibition reverses VILI-induced neural injury of the amygdala and hippocampus and associated acute delirium- and anxiety-like neuropsychiatric functional impairments. These data provide a pathophysiological basis for the development of acute neuropsychiatric impairments following VILI and serve as preclinical justification to assess the effects of IL-6 inhibition as a potential therapeutic intervention to mitigate these highly morbid psychiatric sequelae of VILI.

Prior imaging studies have reported evidence of amygdalar and hippocampal atrophy in individuals with neuropsychiatric impairments [59–61]. Although our results indeed suggest that ongoing cerebral changes during VILI could lead to neural atrophy of the amygdala and hippocampus, the lack of irreversible neural death, as measured by TUNEL staining in this short-term model of VILI, may indicate a window of opportunity to reverse cerebral injury that underlies acute neuropsychiatric impairments after VILI.

While IL-6 is well known to be up-regulated in VILI [17, 18, 32, 34, 46], prior studies have also implicated increased IL-6 signaling in the pathogenesis of diverse neuropsychiatric impairments including, but not limited to, post-traumatic stress disorder, depression, bipolar disorder, and psychotic disorders, each of which may contribute to the risk of acute exacerbation of VILI-induced neuropsychiatric impairments [16, 62–65]. IL-6 is also commonly elevated in acute encephalopathy, a clinical syndrome that itself can contribute to the development of anxiety-like states via fragmented memories and impaired cognitive processing of distressing experiences [20, 21]. As such, as a marker of neural changes in the amygdala and hippocampus, IL-6 mediated injury may contribute diffuse risk to several neuropsychiatric states. Our interest in the relationship of IL-6 and acute neuropsychiatric impairments is based on the prevalence of these conditions in the VILI population, the upregulation of IL-6 in both VILI and acute neuropsychiatric conditions, as well as the well-established involvement of the amygdala and hippocampus in the etiology of neuropsychiatric impairments. Overall, IL-6 signaling may represent a molecular pathway that is common to the pathogenesis of VILI, delirium-, and anxiety-like behaviors, and explain why these neuropsychiatric impairments occur so commonly as a sequela of VILI.

The results of this study suggest that the IL-6 *trans*-signaling pathway mediates acute neuropsychiatric phenotypes after VILI. The IL-6 *trans*-signaling pathway is implicated in the pathogenesis of various neuropsychiatric conditions, including Alzheimer’s disease, Parkinson’s disease, and acute UTI-induced delirium-like states [16, 46, 66]. In IL-6 *trans*-signaling, IL-6 complexes with the soluble IL-6 receptor in the peripheral circulation and directly induces neuronal injury via the gp130 protein. The results of our study provide additional support for this pathway in inducing acute neuropsychiatric impairments after VILI as systemic inhibition of IL-6 ameliorated these impairments. These data are consistent with the substantial clinical and epidemiological data that associate increased systemic IL-6 levels with acute neuropsychiatric phenotypes after lung injury while further suggesting a direct pathological role of IL-6 in inducing these impairments. However, it is also possible that VILI induces acute neuropsychiatric phenotypes via altered activation of cannabinoid or tryptophan pathways, which are known to modulate peripheral and central inflammation, serotonin generation, and neuropsychiatric function [67, 68]. Future studies are thus needed to determine whether VILI effects on acute neuropsychiatric functions are mediated via alterations in cannabinoid or tryptophan pathways.

In this study, CC3 was used as a neuronal injury marker after VILI. Caspase-3 is a known mediator of neuronal apoptosis, which when cleaved to CC3 is considered among the final steps leading to cell death [69]. However, additional pathological roles for CC3 have been described in acute brain ischemia and diverse neurodegenerative conditions [70, 71]. For instance, caspase-3 has been hypothesized to contribute to cognitive dysfunction by cleaving presenilin and promoting generation of amyloid-β, a key peptide implicated in the pathogenesis of Alzheimer’s disease. As such, while our study has focused on the role of CC3 as an apoptotic mediator, it is possible that non-apoptotic pathways also mediate acute neuropsychiatric impairments—these putative mechanisms should be explored in future studies.

There are notable limitations of this study to consider. Although the 35 cc/kg tidal volume is a previously published model of VILI-induced neural injury [7], the clinical relevance of this supraphysiological tidal volume may be limited. However, the heterogeneity of the many potential etiologies that could lead to mechanical ventilation and VILI likely renders any one model insufficient to reflect the entire phenotypic spectrum of VILI-induced neuropathology—nevertheless, future studies using alternative models of lung injury are indicated. In contrast, an important strength of our model is the lack of any other precipitating cause of lung injury or concurrent systemic illness that may confound the relationship between VILI and acute neuropsychiatric impairments. This feature of our model allows the data to demonstrate that even a short duration of mechanical ventilation resulting in mild isolated VILI followed by a brief recovery period precipitates structural and functional phenotypes of acute neuropsychiatric impairments. A final limitation is that we performed behavioral assessments in female mice only as male mice subjected to VILI do not participate in the behavioral assessments due to reduced levels of activity despite no significant differences in PMNs in the BAL or CC3 expression in the amygdala or hippocampus (Additional file 1: Fig. S5). While beyond the scope of this current study, future research is ongoing and necessary to understand whether male mice have increased susceptibility to more severe hypoactive delirium-like phenotypes, consistent with human studies, and the underlying biological mechanisms [72, 73].

In summary, this study provides first evidence of potentially reversible systemic IL-6-mediated neural injury in the amygdala and hippocampus with corresponding acute delirium- and anxiety-like neuropsychiatric functional impairments in a murine model of VILI. These findings provide a pathophysiological basis for acute neuropsychiatric impairments following VILI and call for future studies to assess the effects of IL-6 inhibition or modulation of IL-6 signaling pathways to ameliorate these highly morbid neuropsychiatric conditions that are known to commonly occur following VILI.

## Supplementary Information

**Additional file 1: Table S1.** Antibodies used for immunohistochemistry. **Fig. S1.** No evidence of hypoxic ischemic injury in our VILI model demonstrated by the lack of hypoxic ischemic factor-1α (HIF-1 α) staining in the amygdala compared to an acute ischemic stroke positive control model. **Fig. S2.** Correlations between lung inflammation, amygdalar cytokine concentrations, and CC3. **A**–**B** include SB and VILI animals only while **C–D** include animals from SB, VILI+Saline, VILI+α-IL-6, and VILI+α-IL-6R. **A** No significant correlations between %PMNs in BALF and amygdalar IL-1β or between amygdalar IL-1β and amygdalar CC3. **B** No significant correlation between %PMNs in BALF and hippocampal IL-1β. Direct and significant correlation between hippocampal IL-1β and hippocampal CC3. **C** No significant correlations between %PMNs in BALF and amygdalar IL-1β or between amygdalar IL-1β and amygdalar CC3. **D** No significant correlations between %PMNs in BALF and hippocampal IL-1β or between hippocampal IL-1β and hippocampal CC3. **Fig. S3.** A partial response to the administration α-IL-6 immediately after the completion of ventilation. Independent sample *t* tests revealed **A** no significant difference in amygdalar cleaved caspase-3 (CC3) between saline and α-IL-6 treated males. **B** However, in the hippocampus there was a trend toward significance, with α-IL-6 treated animals have lower CC3. These results indicate early treatment is necessary for the observed effect in both brain regions. *N* = 5–6 per group and data are expressed in mean ± SD. *p<0.05. **Fig. S4.** These data show no significant differences in **A** total distance traveled or **B** total time immobile between the VILI+Saline and VILI+α-IL-6 groups, indicating that overall level of activity did not explain the differences in behavioral function between the two groups. **Fig. S5.** Independent sample t tests indicated no significant differences between male (*n* = 3) and female (*n* = 3) mice after ventilation-induced acute lung injury (VILI) for **A** %PMNs in BALF, **B** amygdalar cleaved caspase-3 (CC3), and **C** hippocampal CC3. These results were observed despite male mice not participating in the behavioral assessments due to reduced levels of activity following VILI (results not shown). Data are expressed in mean ± SD.

## Data Availability

The data that support the findings of this study are available from the corresponding author upon reasonably request.

## Abbreviations

SARS-CoV-2: Severe acute respiratory syndrome coronavirus-2
VILI: Ventilator-induced lung injury
IL-6: Interleukin-6
PMN: Polymorphonuclear
HIF-1α: Hypoxia-inducible factor-1α
CC3: Cleaved caspase-3
SB: Spontaneously breathing
BALF: Bronchoalveolar lavage fluid
TUNEL: Terminal deoxynucleotidyl transferase DUTP nick end labeling
HSP90: Heat-shock protein-90
IL-6R: IL-6-receptor
