# Supplementary material for: Systemic interleukin-6 inhibition ameliorates acute neuropsychiatric phenotypes in a murine model of acute lung injury
Source: Crit Care. 2022 Sep 13;26:274. doi: 10.1186/s13054-022-04159-x (PMC9469063; doi:10.1186/s13054-022-04159-x)
Supplement: Supplementary file 1 — Additional file 1: Table S1. Antibodies used for immunohistochemistry. Fig. S1. No evidence of hypoxic ischemic injury in our VILI model demonstrated by the lack of hypoxic ischemic factor-1α (HIF-1 α) staining in the amygdala compared to an acute ischemic stroke positive control model. Fig. S2. Correlations between lung inflammation, amygdalar cytokine concentrations, and CC3. A–B include SB and VILI animals only while C–D include animals from SB, VILI+Saline, VILI+α-IL-6, and VILI+α-IL-6R. A No significant correlations between %PMNs in BALF and amygdalar IL-1β or between amygdalar IL-1β and amygdalar CC3. B No significant correlation between %PMNs in BALF and hippocampal IL-1β. Direct and significant correlation between hippocampal IL-1β and hippocampal CC3. C No significant correlations between %PMNs in BALF and amygdalar IL-1β or between amygdalar IL-1β and amygdalar CC3. D No significant correlations between %PMNs in BALF and hippocampal IL-1β or between hippocampal IL-1β and hippocampal CC3. Fig. S3. A partial response to the administration α-IL-6 immediately after the completion of ventilation. Independent sample t tests revealed A no significant difference in amygdalar cleaved caspase-3 (CC3) between saline and α-IL-6 treated males. B However, in the hippocampus there was a trend toward significance, with α-IL-6 treated animals have lower CC3. These results indicate early treatment is necessary for the observed effect in both brain regions. N = 5–6 per group and data are expressed in mean ± SD. *p<0.05. Fig. S4. These data show no significant differences in A total distance traveled or B total time immobile between the VILI+Saline and VILI+α-IL-6 groups, indicating that overall level of activity did not explain the differences in behavioral function between the two groups. Fig. S5. Independent sample t tests indicated no significant differences between male (n = 3) and female (n = 3) mice after ventilation-induced acute lung injury (VILI) for A %PMNs in BALF [file 13054_2022_4159_MOESM1_ESM.docx]

**Systemic Interleukin-6 Inhibition Ameliorates Acute Neuropsychiatric Phenotypes in A Murine Model of Acute Lung Injury**

Faizan Anwar, BS, Nicklaus A. Sparrow, PhD, Mohammad Harun Rashid, PhD, Gena Guidry, MS, Michael M. Gezalian, MD, Eric J. Ley, MD, Maya Koronyo-Hamaoui, PhD, Itai Danovitch, MD, E. Wesley Ely, MD, S. Ananth Karumanchi, MD, Shouri Lahiri, MD

Online Data Supplement

**Supplementary Table 1**

| **Antibodies Used** | | | |
| --- | --- | --- | --- |
| **Antigen** | **Dilution** | **Vendor** | **Catalog #** |
| *c-fos* | 500 | Santa Cruz | SC-52 |
| Cl. Caspase-3 | 800 | Cell Sig. | 9664 |
| HIF-1α | 600 | Millipore | MAB5382 |
| HSP90 | 300 | Life Tech. | 37-9400 |
| IL-1β | 150 | Cell Sig. | 12242S |
| IL-6 | 150 | Cell Sig. | 12912S |
| IL-6 | 150 | Life Tech. | AMC0864 |
| TNF-α | 150 | Cell Sig. | 11948S |

Antibodies used for immunohistochemistry

**Supplementary Figure 1**


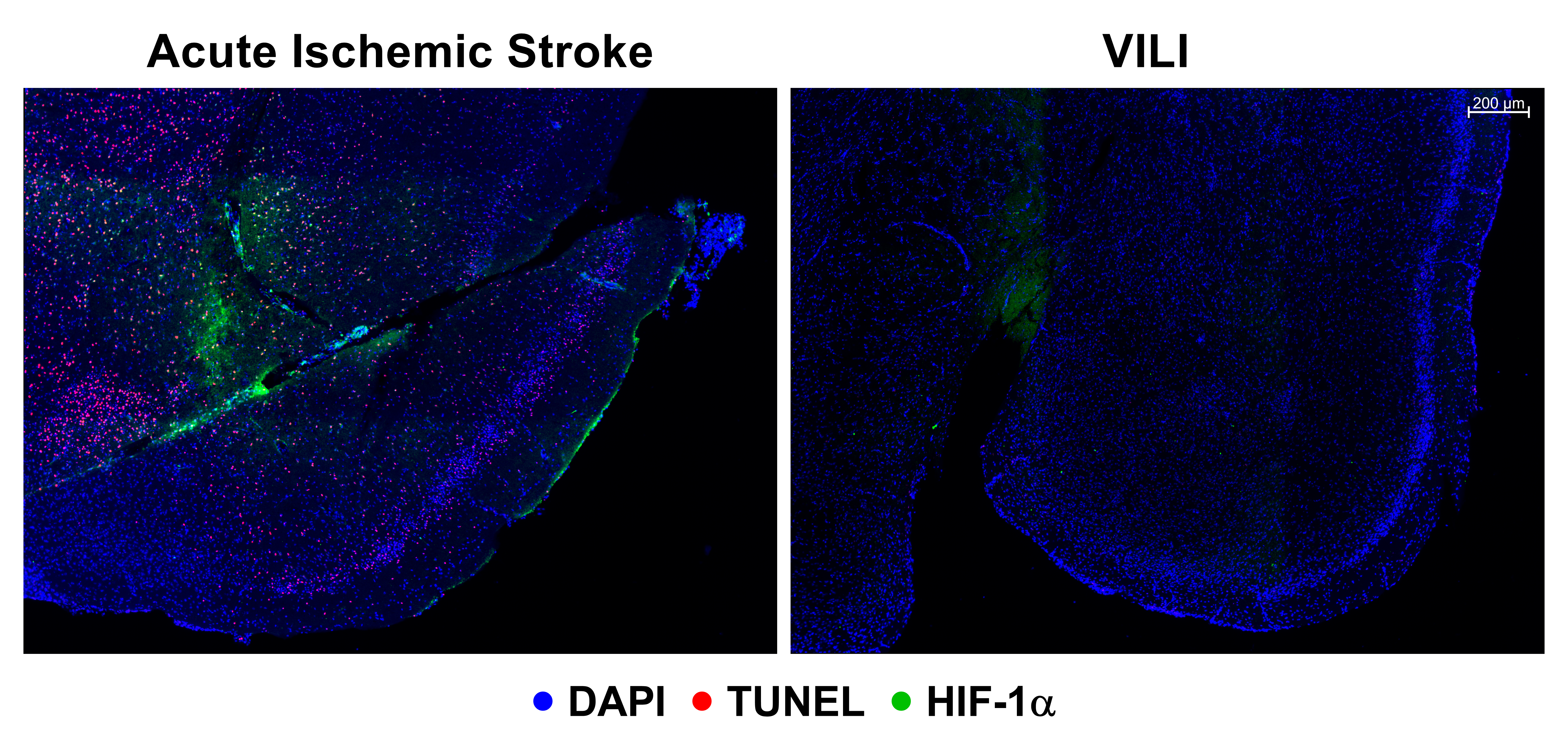


No evidence of hypoxic ischemic injury in our VILI model demonstrated by the lack of hypoxic ischemic factor-1α (HIF-1 α) staining in the amygdala compared to an acute ischemic stroke positive control model.

**Supplementary Figure 2**


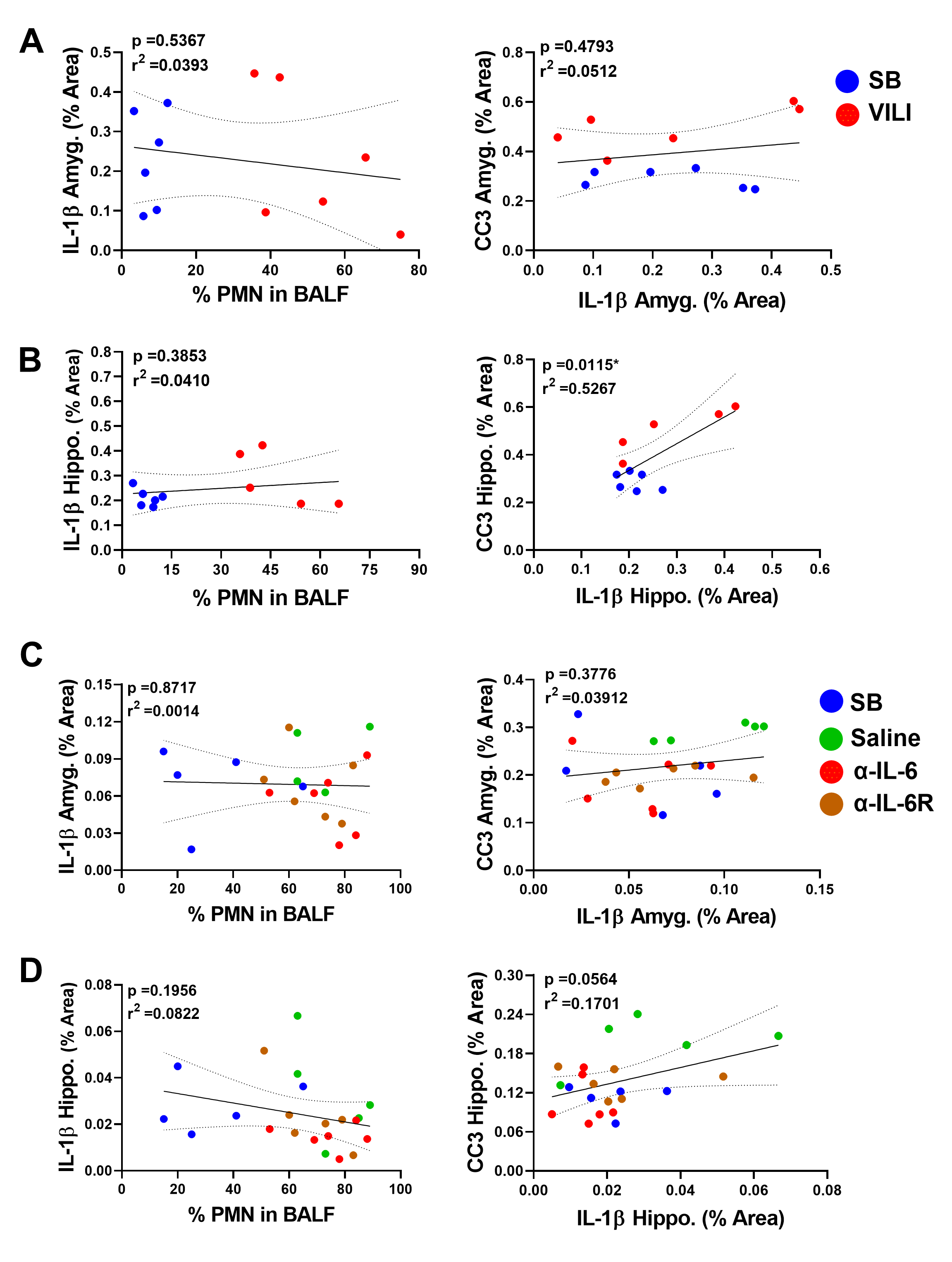


Correlations between lung inflammation, amygdalar cytokine concentrations, and CC3. A-B include SB and VILI animals only while C-D include animals from SB, VILI+saline, VILI+α-IL-6, and VILI+α-IL-6R.

A: No significant correlations between %PMNs in BALF and amygdalar IL-1β or between amygdalar IL-1β and amygdalar CC3. B: No significant correlation between %PMNs in BALF and hippocampal IL-1β. Direct and significant correlation between hippocampal IL-1β and hippocampal CC3. C: No significant correlations between %PMNs in BALF and amygdalar IL-1β or between amygdalar IL-1β and amygdalar CC3. D: No significant correlations between %PMNs in BALF and hippocampal IL-1β or between hippocampal IL-1β and hippocampal CC3.

**Supplementary Figure 3**


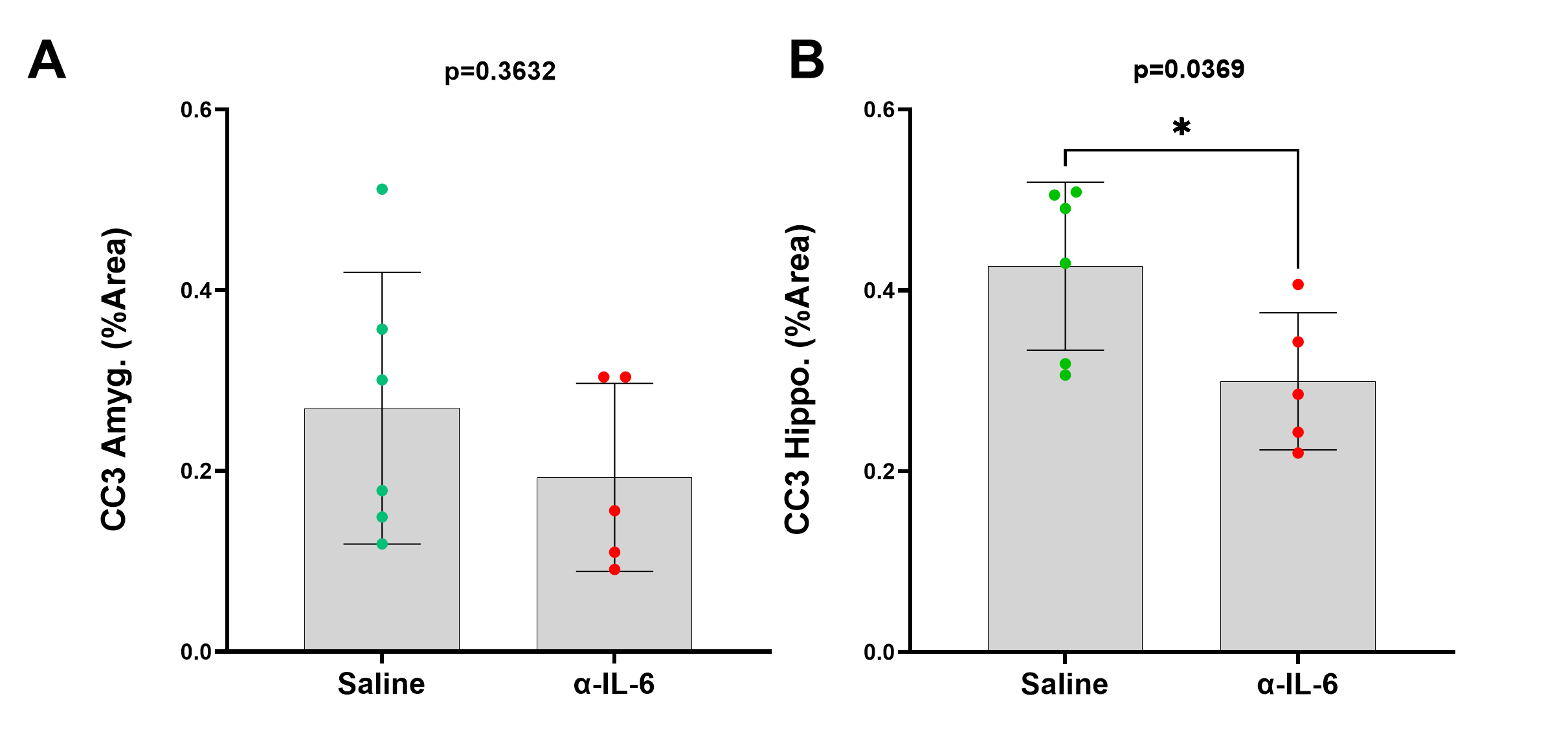


A partial response to the administration α-IL-6 immediately after the completion of ventilation. Independent sample t-tests revealed A: no significant difference in amygdalar cleaved caspase-3 (CC3) between saline and α-IL-6 treated males. B: However, in the hippocampus there was a trend toward significance, with α-IL-6 treated animals have lower CC3. These results indicate early treatment is necessary for the observed effect in both brain regions. N = 5-6 per group and data are expressed in mean ± SD. *p<0.05.

**Supplementary Figure 4**


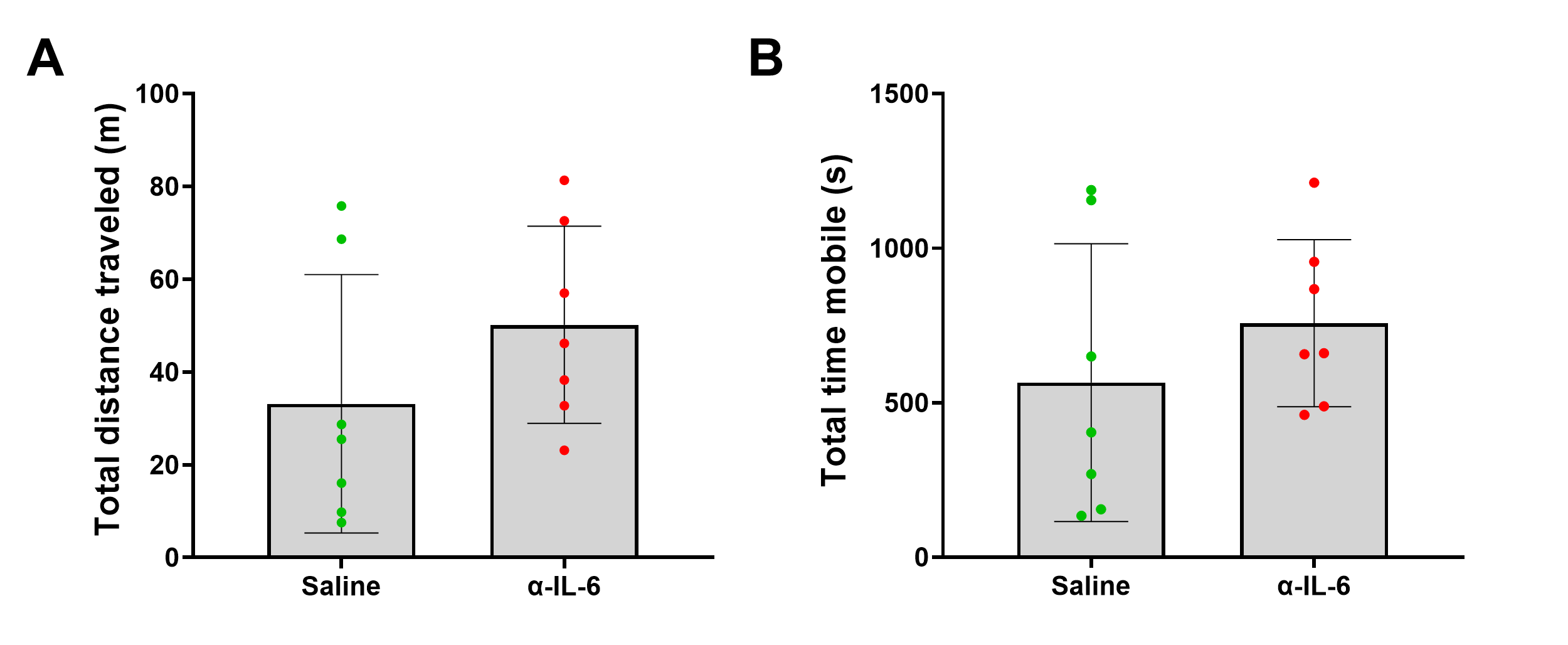


These data show no significant differences in A: total distance traveled or B: total time immobile between the VILI+saline and VILI+α-IL-6 groups, indicating that overall level of activity did not explain the differences in behavioral function between the two groups.

*Supplementary Figure 5*


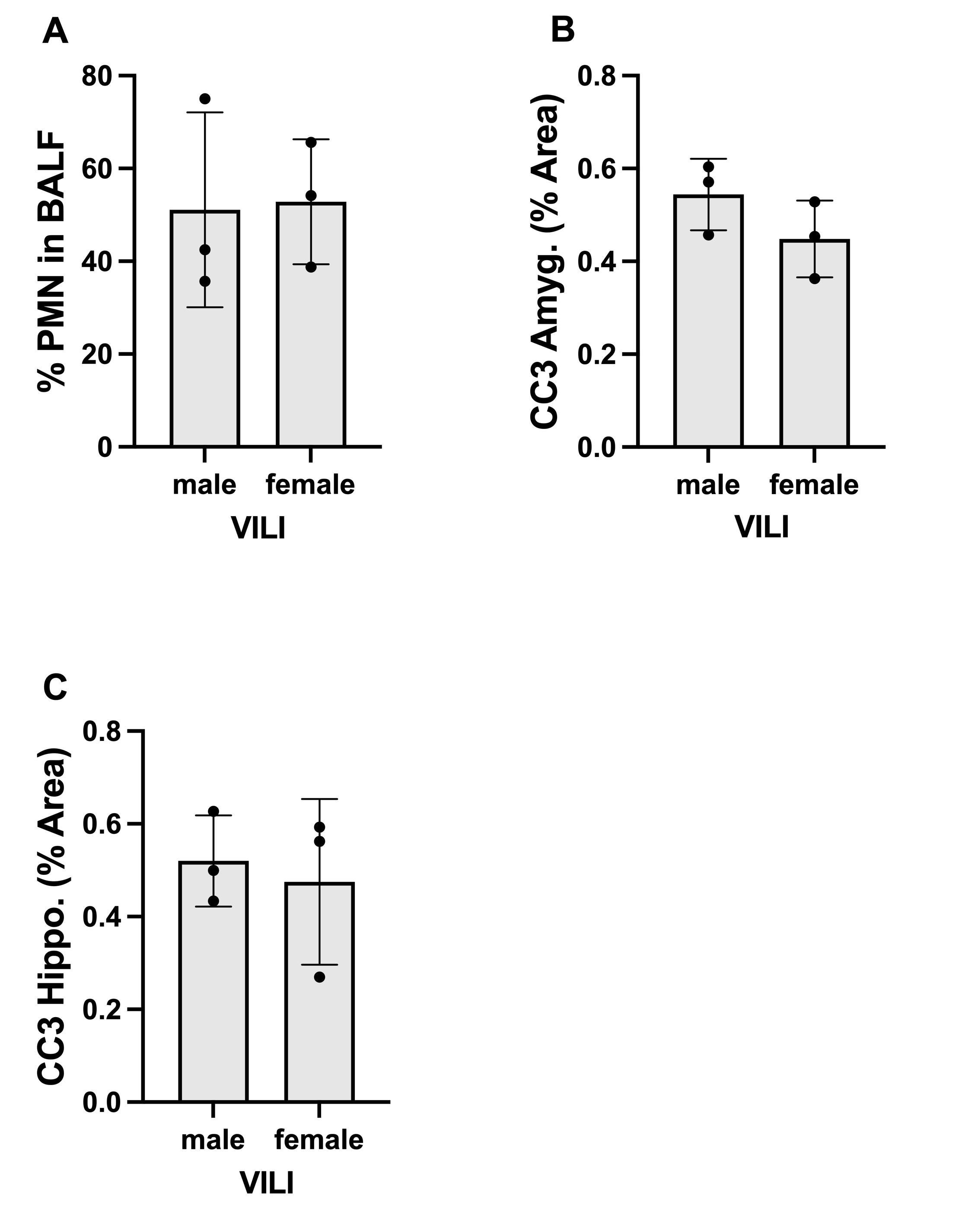


Independent sample t-tests indicated no significant differences between male (n =3) and female (n = 3) mice after ventilation-induced acute lung injury (VILI) for A: %PMNs in BALF, B: amygdalar cleaved caspase-3 (CC3), and C: hippocampal CC3. These results were observed despite male mice not participating in the behavioral assessments due to reduced levels of activity following VILI (results not shown). Data are expressed in mean ± SD.
